# Carbamate Pesticide-Induced Apoptosis in Human T Lymphocytes

**DOI:** 10.3390/ijerph120403633

**Published:** 2015-04-01

**Authors:** Qing Li, Maiko Kobayashi, Tomoyuki Kawada

**Affiliations:** Department of Hygiene and Public Health, Nippon Medical School, Tokyo 113-8602 Japan; E-Mails: mk831111@nms.ac.jp (M.K.); kawada@nms.ac.jp (T.K.)

**Keywords:** annexin-V, apoptosis, carbamate pesticide, carbaryl, caspase, cytochrome-c, Jurkat T cell, maneb, thiram, ziram

## Abstract

We previously found that carbamate pesticides induced significant apoptosis in human natural killer cells. To investigate whether carbamate pesticides also induce apoptosis in human T lymphocytes, in the present study Jurkat human T cells were treated *in vitro* with thiram, maneb, carbaryl or ziram. Apoptosis was determined by FITC-Annexin-V/PI staining. To explore the mechanism of apoptosis, intracellular levels of active caspase 3 and mitochondrial cytochrome-c release were determined by flow cytometry. We found that thiram, ziram, maneb and carbaryl also induced apoptosis in a time- and dose-dependent manner in the human T cells. However, the strength of the apoptosis-inducing effect differed among the pesticides, with the: thiram > ziram > maneb > carbaryl. Moreover, thiram significantly increased the intracellular level of active caspase 3 and caspase inhibitors significantly inhibited apoptosis. Thiram also significantly caused mitochondrial cytochrome-c release. These findings indicate that carbamate pesticides can induce apoptosis in human T cells, and the apoptosis is mediated by the activation of caspases and the release of mitochondrial cytochrome-c.

## 1. Introduction

Carbamate pesticides are widely used in agriculture throughout the world as fungicides and insecticides [1,2,3,4]. It has been reported that exposure to carbamate pesticides statistically significantly increased risk of non-Hodgkin’s Lymphoma in humans [5], suggesting that carbamate pesticides may cause impairments of human immune system because natural killer (NK) cells and cytotoxic T lymphocytes (CTL) provide host defense against tumors. Based on the above background, we previously investigated the effect of carbamate pesticides on NK cells and found that carbamate pesticide significantly inhibited human NK activity by reductions of intracellular perforin, granzyme A, granzyme B, granzyme 3/K, and granulysin levels in NK cells [6,7]. We previously also found that ziram significantly induced apoptosis in human T lymphocytes, NK cells and monocytes [8,9,10] and contributed to the inhibition of NK and CTL activity. Recently we found that several other carbamate pesticides also induced apoptosis in human NK cells [11]. However, it is not clear whether other carbamate pesticides can induce apoptosis in human T lymphocytes. Thus, in the present study, we selected carbaryl (insecticide), maneb (fungicide) and thiram (fungicide) [1,2,3], which are used now in Japan as pesticides and investigated their apoptosis-inducing ability in human T cells although the three pesticides have different chemical structures and different modes of action as plant protection products [1,2,3].

Apoptosis was originally proposed by Kerr in 1972 as a form of programmed cell death in multicellular organisms, and involves a series of biochemical events leading to a characteristic cell morphology and death, including blebbing, changes to the cell membrane, such as loss of membrane asymmetry and attachment, cell shrinkage, nuclear fragmentation, chromatin condensation, and chromosomal DNA fragmentation [12,13]. Apoptosis in the early stage can be detected by fluorescein isothiocynate (FITC)-annexin-V staining using a flow cytometer [8,9,10,11].

The caspase family of cysteine proteases plays a key role in apoptosis. Caspase-3 is a key protease activated during the early stages of apoptosis and, like other members of the caspase family, is synthesized as an inactive proenzyme that is processed in cells undergoing apoptosis by self-proteolysis and/or cleavage by another protease such as caspase 8 or 9 [14,15]. Cytochrome-c initiates apoptosis through its release into the cytoplasm and binding of Apaf-1 which activates procaspase 9 [16]. Goldstein *et al.* [17] reported that the release of cytochrome-c from mitochondria was a very early event during apoptosis. Based on this background, we also investigated the effects of carbamate pesticides on caspases and cytochrome-c release to explore the mechanism of the apoptosis. 

## 2. Materials and Methods

### 2.1. Reagents

RPMI 1640 medium was purchased from GIBCO (Carlsbad, CA, USA). Fetal bovine serum (FBS) was purchased from JRH Biosciences (Lenexa, KS, USA), and heat-inactivated at 56 °C for 30 min prior to use. Glutamine, 2-mercaptoethanol (2-ME) and propidium iodide (PI) were obtained from Sigma (St. Louis, MO, USA). Fluorescein isothiocynate (FITC)-anti human annexin V and FITC-anti human active caspase-3, Z-DEVD-FMK (a caspase-3 inhibitor), Z-VAD-FMK (a general caspase inhibitor), Z-FA-FMK (a negative control for Z-DEVD-FMK and Z-VAD-FMK), and Cytofix/cytoperm solution were purchased from BD Pharmingen (San Diego, CA, USA). Carbaryl, maneb, thiram and ziram were obtained from Wako Pure Chemical Industries (Osaka, Japan) and prepared as stock solutions in DMSO. 

### 2.2. Cells

The human Jurkat T cell line was obtained from American Type Culture Collection (ATCC) (Manassas, VA, USA) and maintained in RPMI 1640 medium containing 10% FBS [9]. 

### 2.3. Carbamate Pesticide-Induced Apoptosis in Jurkat T Cells Determined by FITC-Annexin V/PI Staining

Jurkat T cells at 1 × 10^5^ /mL were treated with carbaryl, maneb, thiram or ziram at 0-40 μM for 4, 8, 16 or 24 h at 37 °C in a 5% CO_2_ incubator. The treated cells were stained with FITC-annexin-V/PI, and 10,000 cells were acquired and stored for analysis with a FACScan flow cytometer (Becton Dickinson, San Jose, CA, USA) as described previously [8,9,10,11]. Apoptotic cells were defined as higher FITC-annexin-V/low PI and the late apoptotic cells were defined as higher FITC-annexin-V/ higher PI.

### 2.4. Determination of Intracellular Levels of Active Caspase-3 in Jurkat Cells by Flow Cytometry

Jurkat T cells at 1 × 10^5^ /mL were incubated with thiram at 0 (0.1% DMSO), 0.0625, 0.125, 0.25, 0.5 or 1 µM for 16 h at 37 °C in a 5% CO_2_ incubator, harvested, and washed twice with PBS. The cells were fixed/permeablized with Cytofix/cytoperm solution for 20 min at 4 °C, and active caspase-3 was stained with FITC-anti human active caspase-3 for 30 min at room temperature according to the manufacturer’s instructions (BD PharMingen). Again, the flow cytometric analysis was performed with FACScan (10,000 cells per analysis) [8,9,10,11].

### 2.5. Protective Effects of Caspase-3 and General Caspase Inhibitors against Thiram-Induced Apoptosis in Jurkat T Cells

Jurkat T cells at 1 × 10^5^ /mL were preincubated with Z-DEVD-FMK, an inhibitor of caspase-3, Z-VAD-FMK, a general caspase inhibitor, or Z-FA-FMK, a negative control for Z-DEVD-FMK and Z-VAD-FMK, at 20 µM for 30 min, treated with thiram at 0 (0.1% DMSO), 0.125, or 0.5µM for 16 h, harvested, and washed twice with PBS. The treated cells were stained with FITC-Annexin-V/PI. Flow cytometric analysis was performed with FACScan (10,000 cells for each analysis) [8,9,10,11].

### 2.6. Analysis of Cytochrome-c Release

Jurkat cells at 1x10^5^ /mL were incubated with thiram at 0 (0.1% DMSO), 0.0625, 0.125, 0.25, 0.5, or 1 µM for 16 h and 24h at 37 °C in a 5% CO_2_ incubator, harvested, and washed twice with PBS. The cells were fixed/permeablized with Cytofix/cytoperm solution for 20 min at 4 °C, and the intracellular cytochrome-c was stained with FITC-anti human cytochrome-c (mouse IgG1) or FITC-mouse IgG1 as an isotypic control for 30 min at 4 °C according to the manufacturer’s instructions (eBioscience, San Diego, CA, USA). Flow cytometric analysis was performed with FACScan (10,000 cells per analysis) [8,9,10,11,18].

### 2.7. Statistical Analyses

The numbers of apoptotic cells, active caspase-positive cells and cytochrome-c-negative cells were used for statistical analyses. Statistical analyses were performed using one-way ANOVAs followed by a post hoc test, Tukey’s test, with SPSS 16.0J software for Windows. Paired and unpaired t-tests were also conducted. The significance level for *p*-values was set at < 0.05.

## 3. Results

### 3.1. Thiram-Induced Apoptosis in Jurkat Cells Determined by FITC-Annexin-V/PI Staining

**Figure 1:**
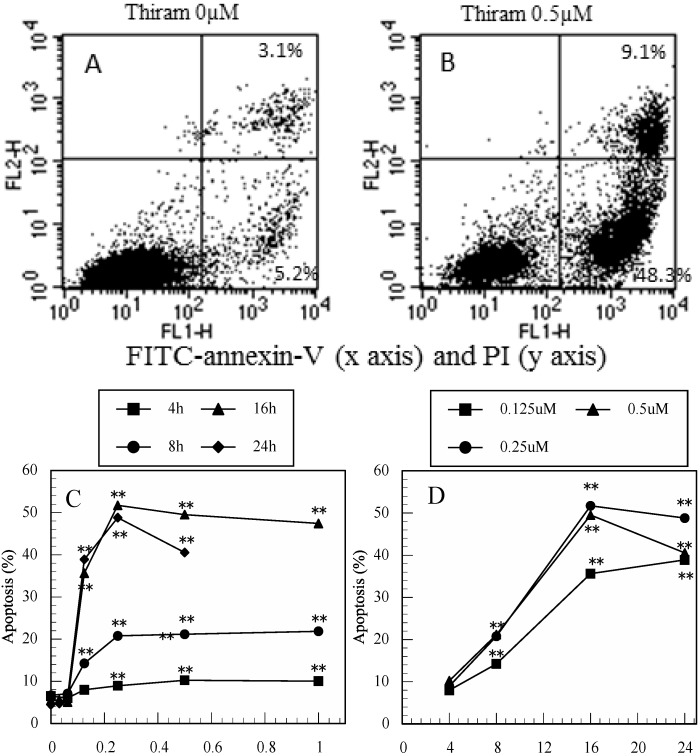

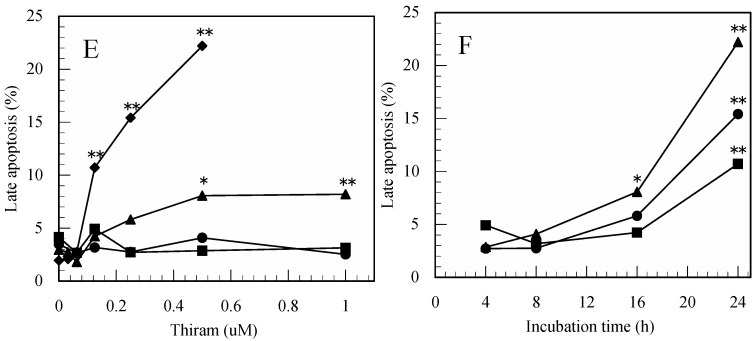
Thiram induced apoptosis in Jurkat T cells. (**A**): dot plot of FITC-annexin V (x axis)/PI (y axis) in control cells, (**B**): dot plot of FITC-annexin V/PI in thiram-treated cells at 16 h, percentages in quadrants 2 and 3 show FITC-annexin V^+^/PI^+^ (late apoptosis) and FITC-annexin V^+^/PI^−^ (apoptosis) cells, respectively. (**C**): dose-dependent increases in apoptotic cells in thiram-treated cultures, (**D**): time-dependent increases in apoptotic cells in thiram-treated cultures, (**E**): dose-dependent increases in late apoptotic cells in thiram-treated cultures, (**F**): time-dependent increases in late apoptotic cells in thiram-treated cultures. Data are presented as the mean (n = 3). One-way ANOVA indicated that both the concentration of thiram and period of incubation significantly affected apoptosis (all *p* < 0.01) and late apoptosis (*p* < 0.01 for 16 and 24 h). *: *p* < 0.05, **: *p* < 0.01, significantly different from 0 µM (Figure 1C,E) or from 4 h (Figure 1D,F) by Tukey’s test.

As shown in Figure 1A,B, 48.3% of thiram-treated cells exhibited apoptosis (FITC-Annexin-V^+^/PI^−^) and 9.1% showed late apoptosis (FITC-Annexin-V^+^/PI^+^) (Figure 1B, 16h), compared to only 5.2% and 3.1% of control cells, respectively (Figure 1A, 16h). As shown in Figure 1C,D, thiram induced apoptosis in a dose- and time-dependent manner. Similarly, as shown in Figure 1E,F, thiram-induced late apoptosis also exhibited a dose- and time-dependent profile.

### 3.2. Maneb-Induced Apoptosis in Jurkat T Cells Determined by FITC-Annexin-V/PI Staining

**Figure 2:**
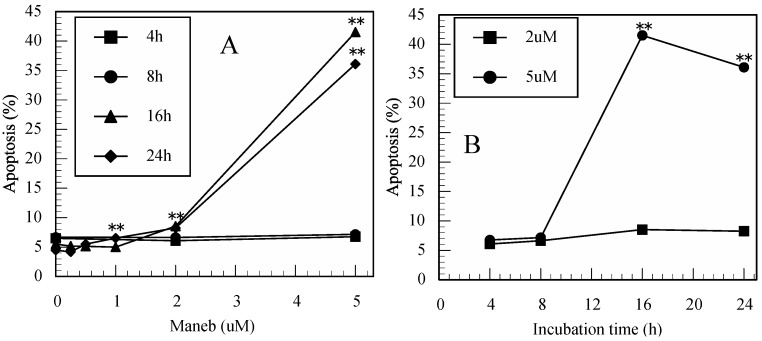

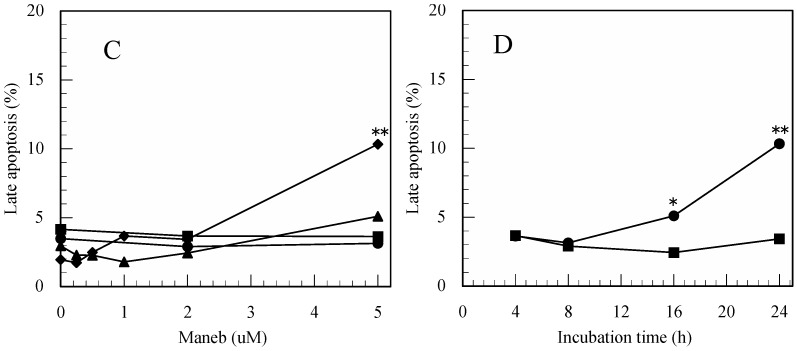
Maneb induced apoptosis in Jurkat T cells. (**A**): dose-dependent increases in apoptotic cells in maneb-treated cultures, (**B**): time-dependent increases in apoptotic cells in maneb-treated cultures, (**C**): dose-dependent increases in late apoptotic cells in maneb-treated cultures, (**D**): time-dependent increases in late apoptotic cells in maneb-treated cultures. Data are presented as the mean (n = 3). One-way ANOVA indicated that both the concentration of maneb for 16 and 24 h and period of incubation with 5 µM significantly affected apoptosis and late apoptosis (all *p* < 0.01). *: *p* < 0.05, **: *p* < 0.01, significantly different from 0 µM (Figure 2A,C) or from 4 h (Figure 2B,D) by Tukey’s test.

As shown in Figure 2A–D, maneb also induced cell death (apoptosis and late apoptosis) in a dose- and time-dependent manner in Jurkat T cells. However, the concentrations were higher than for thiram. 

### 3.3. Carbaryl-Induced Apoptosis in Jurkat T Cells Determined by FITC-Annexin-V/PI Staining

**Figure 3:**
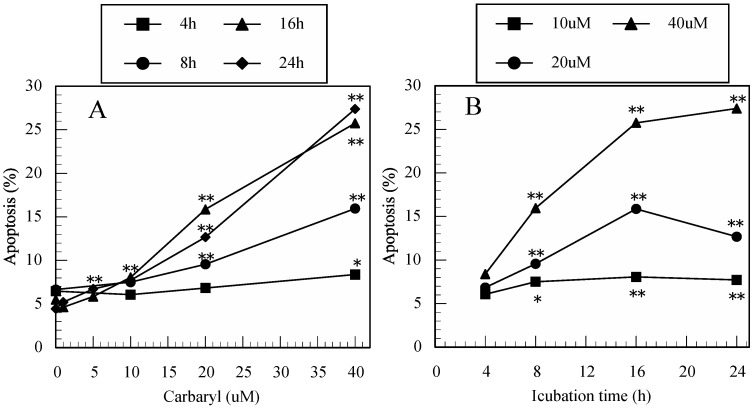

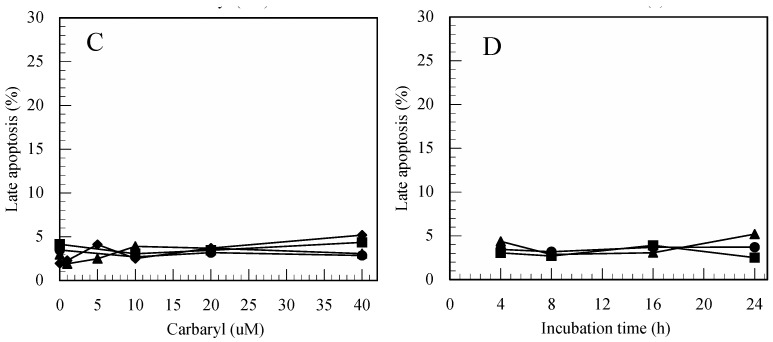
Carbaryl induced apoptosis in Jurkat T cells. (**A**): dose-dependent increases in apoptotic cells in carbaryl-treated cultures, (**B**): time-dependent increases in apoptotic cells in carbaryl-treated cultures, (**C**): dose-effect in late apoptotic cells in carbaryl-treated cultures, (**D**): time-course in late apoptotic cells in carbaryl-treated cultures. Data are presented as the mean (n = 3). One-way ANOVA indicated that both the concentration of carbaryl and period of incubation significantly affected apoptosis (all *p* < 0.01), but did not affect late apoptosis. *: *p* < 0.05, **: *p* < 0.01, significantly different from 0 µM (Figure 3A,C) or from 4 h (Figure 3B,D) by Tukey’s test.

As shown in Figure 3A,B, carbaryl also induced apoptosis in a dose- and time-dependent manner in Jurkat T cells; however, the concentrations were higher than for thiram and maneb. However, as shown in Figure 3C,D, carbaryl did not induce late apoptotosis even at a very high concentration (40 µM).

### 3.4. Difference in the Ability to Induce Apoptosis among the Pesticides

**Figure 4:**
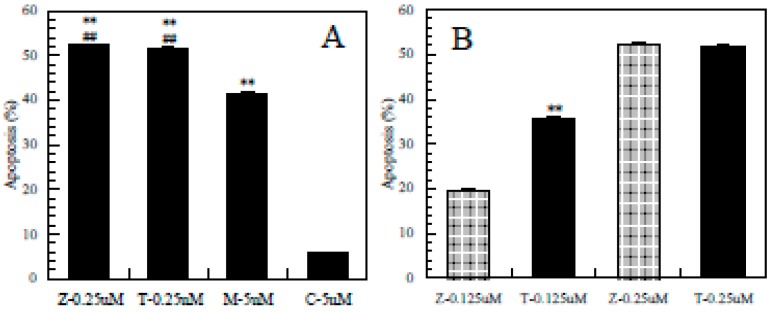
Difference in the ability to induce apoptosis among the pesticides. Z-0.25µM = ziram at 0.25µM, Z-0.125 µM = ziram at 0.125 µM; T-0.25µM = thiram at 0.25µM, T-0.125 µM = thiram at 0.125 µM; M-5µM = meneb at 5 µM; C-5µM = carbaryl at 5 µM, **: *p* < 0.01, significantly different from 5 µM of carbaryl, ##: *p* < 0.01, significantly different from 5 µM of maneb (**A**), **: *p* < 0.01 significantly different from 0.125 µM of ziram (**B**) by t-test.

As shown in Figure 4A, the apoptosis induced by ziram and thiram at 0.25 µM was significantly greater than that induced by maneb and carbaryl at 5 µM, respectively; moreover, the apoptosis induced by maneb at 5 µM was significantly greater than that induced by carbaryl at 5 µM. 

As shown in Figure 4B, although thiram and ziram had almost the same effect at 0.25 µM, the apoptosis induced by thiram at 0.125 µM was significantly greater than that induced by ziram at 0.125 µM, indicating that thiram was stronger than ziram. Taken together, the apoptosis-inducing effect differed among these pesticides in the order: thiram > ziram > maneb > carbaryl.

### 3.5. Detection of Intracellular Levels of Active Caspase-3 in Apoptotic Jurkat T Cells by Flow Cytometry

**Figure 5:**
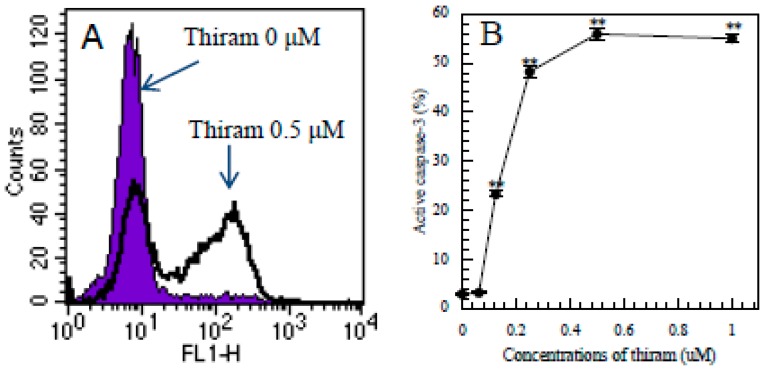
Thiram induced an increase in active caspase-3-positive Jurkat T cells. (**A**): the shaded histogram shows the control cells (thiram at 0 µM) and the open histogram shows the cells treated with thiram at 0.5 µM for 16 h and stained with FITC-rabbit anti-human active caspase 3. (**B**): dose-dependent increases in active caspase-3-positive cells in thiram-treated cultures. Data are presented as the mean ±SD (n = 3). One-way ANOVA indicated that the concentration of thiram significantly affected the active caspase-3-positive cells (*p* < 0.01). **: *p* < 0.01, significantly different from 0 µM by Tukey’s test.

To explore the mechanism of the apoptosis, we investigated whether thiram affected the intracellular level of active caspase-3. As shown in Figure 5A,B, thiram induced a significant increase in active caspase-3 in a dose-dependent manner, suggesting that thiram induced apoptosis at least partially *via* the caspase-3 pathway. 

### 3.6. Protective Effects of Caspase-3 and General Caspase Inhibitors on Thiram-Induced Apoptosis in Jurkat T Cells

**Figure 6:**
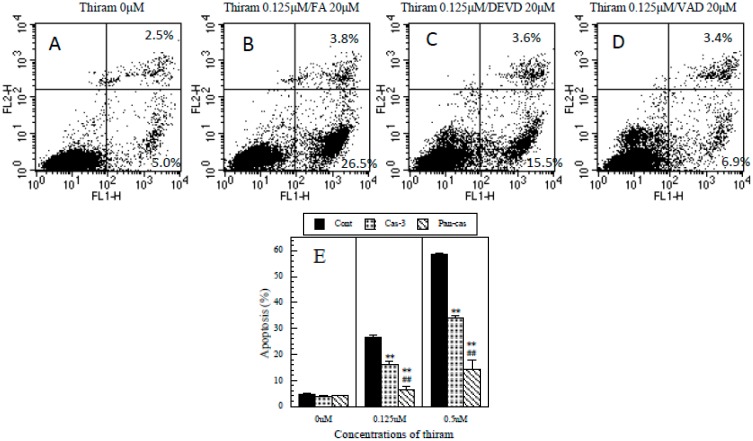
Protective effects of caspase-3 and general caspase inhibitors on thiram-induced apoptosis. (**A**): dot plot of FITC-Annexin V (x axis)/PI (y axis) in control cells (thiram at 0 µM), (**B**): dot plot of FITC-Annexin V/PI in cells treated with thiram at 0.125 µM in the presence of Z-FA-FMK (a negative control for Z-VAD-FMK and Z-DEVD-FMK) at 20 µM, (**C**): dot plot of FITC-Annexin V/PI in cells treated with thiram at 0.125 µM in the presence of Z-DEVD-FMK (a caspase-3 inhibitor) at 20 µM, (**D**): dot plot of FITC-Annexin V/PI in cells treated with thiram at 0.125 µM in the presence of Z-VAD-FMK (a general caspase inhibitor) at 20 µM; percentages in quadrants 2 and 3 show FITC-Annexin V^+^/PI^+^ (late apoptosis) and FITC-Annexin V^+^/PI^−^ (apoptosis) cells, respectively, (**E**) Cont: cells pre-treated with Z-FA-FMK (a negative control) at 20 µM, Cas-3: cells pre-treated with Z-DEVD-FMK (a caspase-3 inhibitor) at 20 µM, Pan-cas: cells pre-treated with Z-VAD-FMK (a general caspase inhibitor) at 20 µM. Data are presented as the mean ±SD (n = 3). **: *p* < 0.01, significantly different from Cont, ##: *p* < 0.01, significantly different from Cas-3 by paired t-test.

As shown in Figure 6, an inhibitor of active caspase-3 partially but significantly protected against the apoptosis induced by thiram. Moreover, a general inhibitor of caspases significantly and almost completely protected against the apoptosis. The findings strongly suggest that thiram induced apoptosis *via* the caspase cascade pathway. 

### 3.7. Detection of Mitochondrial Cytochrome-c Release in Apoptotic Jurkat T Cells by Flow Cytometry

**Figure 7:**
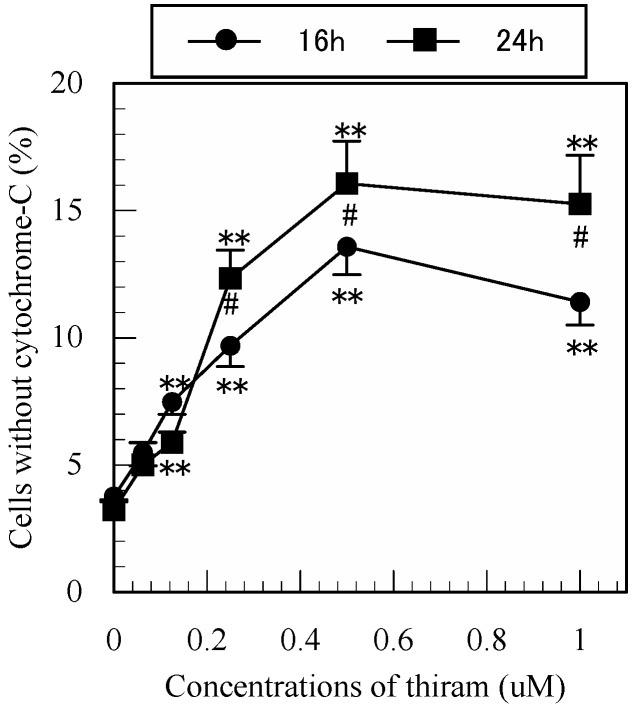
Detection of mitochondrial cytochrome-c release in apoptotic cells by flow cytometry. Data are presented as the mean ±SD (n = 3). **: *p* < 0.01, significantly different from 0 µM, #: *p* < 0.05, significantly different between 16 and 24 h by Tukey’s test.

To further explore the mechanism of thiram-induced apoptosis in Jurkat T cells, we investigated whether thiram induces mitochondrial cytochrome-c release. As shown in Figure 7, thiram induced a significant increase in the number of cytochrome-c-negative cells in a dose-dependent manner at 16 h and 24 h after treatment, indicating that it induced mitochondrial cytochrome-c release. As shown in Table 1, two-way repeated measures ANOVA indicated that both concentration and period of treatment of thiram significantly affected cytochrome-c release (all *p* < 0.01).

**Table 1:** Analytical results of the two-way repeated measures ANOVA.

Factors	Variations	Degrees of Freedom	Variances	Observed Variance Ratios	*p* Values
Period of treatment (h)	10.12	1	10.12	10.11	0.004
Concentration of thiram (µM)	638.74	5	127.75	127.64	0.000
Interactions	36.28	5	7.26	7.25	0.000
Errors	24.02	24	1.00		
Total	709.17	35			

## 4. Discussion

Human exposure to carbamate pesticides may occur by coming into contact with latex rubber, ingesting treated crops, or via inhalation [19]. In Japan, the residual standards for carbamate pesticides in rice, potato and tomato are 0.3, 0.2 and 5 ppm, calculated as carbon disulfide, respectively [20], suggesting the potential of human exposure from crops. In the present study, we used 0.0625–1 µM (approximately 0.015−0.24 ppm) of thiram *in vitro*, which are reasonable related to the possible human exposure. Because maneb and carbaryl showed weaker toxicity than thiram and ziram in T cells higher concentrations of maneb and carbaryl were used in the present study.

Exposure to carbamate pesticides statistically significantly increased risk of non-Hodgkin’s Lymphoma in human [5] suggesting that carbamate pesticides may cause impairments of human immune system because NK and CTL cells provide host defense against tumors. Based on the above background, we investigated the effects of carbamate pesticides on human NK cells and found that ziram, a carbamate pesticide, significantly inhibited human NK and CTL activity [6]. On exploring the mechanism of this inhibition, we found that ziram significantly induces apoptosis/necrosis in human T cells, which contributed to the inhibition of CTL activity [6]. However, it is not clear whether other carbamate pesticides have a similar apoptosis-inducing effect. Thus, in the present study, we tested several carbamate pesticides including carbaryl (insecticide), maneb (fungicide) and thiram (fungicide) [1,2,3], which are used now in Japan as pesticides in human T cells. Similar to ziram [9], thiram induced apoptosis at a very low concentration (0.125 μM) in a dose- and time-dependent manner in Jurkat cells. Moreover, maneb and carbaryl induced apoptosis in a dose- and time-dependent manner at higher concentrations. However, the strength of the apoptosis-inducing effect differed among these pesticides, with the: thiram > ziram > maneb > carbaryl.

To explore the mechanism of thiram-induced apoptosis, we examined the active caspase-3 in thiram-treated Jurkat T cells and found a significant increase in its intracellular levels. Moreover, Z-DEVD-FMK, a caspase-3 inhibitor, significantly inhibited the apoptosis. These findings suggested that thiram induces apoptosis via the caspase-3 pathway. However, the caspase-3 inhibitor only partially prevented apoptosis, suggesting other pathways to be involved. To explore whether other caspases are involved in the apoptosis, we investigated the protective effect of a general caspase inhibitor, Z-VAD-FMK, and found that it significantly and almost completely blocked the thiram-induced apoptosis indicating the involvement of a caspase cascade. We previously found that ziram significantly activated the intracellular caspases 3/7, 8, 9 and pan-caspase in a dose-dependent manner and that caspase-3 and general caspase inhibitors significantly and almost completely blocked the ziram-induced apoptosis in Jurkat T cells [9], suggesting that thiram has a similar mechanism of action to ziram. 

Cytochrome-c initiates apoptosis through its release into the cytoplasm and binding of Apaf-1 which activates procaspase 9 [16]. Goldstein *et al.* [17] reported that the release of cytochrome-c from mitochondria was a very early event during apoptosis. To explore whether this release mechanism was involved in the thiram-induced apoptosis, we determined intracellular cytochome-c levels in Jurkat T cells. Thiram produced a significantly higher proportion of cells without cytochome-c in a dose-dependent manner, indicating that it induced the release of cytochrome-c from mitochondria. This finding also suggested that cytochrome-c was involved in the apoptosis. We previously found that ziram significantly induced the release of cytochrome-c from mitochondria in Jurkat T cells [9], again suggesting that thiram has a similar action mechanism to ziram in inducing apoptosis. Another experiment should be conducted to investigate effects of carbamate pesticides on the oxidative stress, respiratory chain complex and mitochondrial transmembrane potential. Taken together, the above findings suggested that thiram affects the caspase-cascade and the release of mitochondria/cytochrome-c.

The strength of the apoptosis-inducing effect differed among these pesticides, in the order: thiram > ziram > maneb > carbaryl. We speculate that the general toxicity of the chemicals contributed to the difference, and used the LD50 to evaluate this toxicity. The oral LD_50_ values of carbaryl, thiram, ziram and maneb in rats are 250 mg/kg [21], 640 mg/kg [3], 1,400 mg/kg [4] and 2600−5500 mg/kg [2], respectively. Carbaryl shows the strongest acute toxicity, but the weakest apoptosis-inducing effect of these chemicals, suggesting that the two factors are not related. 

Solubility in water may contribute to the difference. The solubility of carbaryl, thiram, ziram and maneb in water is 120 mg/L (120 ppm) [1], 30 mg/L (30 ppm) [3], 65 mg/L (65 ppm) [4] and 20 mg/L (20 ppm) [2], respectively. The highest concentrations of carbaryl, thiram, ziram and maneb used in the present study were 40 μM (8 ppm), 1 μM (0.24 ppm), 1 μM (0.312 ppm) and 5 μM (1.327 ppm), respectively. Thus all the chemicals should have dissolved in the culture medium, suggesting that solubility did not contribute to the difference. The mechanism involved should be explored in a future study. Although these chemicals are rapidly metabolized within several days in animals [1,2,3,4], it is difficult to account for their metabolism *in vitro* where they may bind to the membrane of cells or enter the cells. Another experiment should be conducted to investigate the structure activity relationship of the pesticides and explore the mechanism of the different strength of the apoptosis-inducing effect. Taken together, the present findings indicate that similar to ziram, thiram, maneb and carbaryl also induce apoptosis in human T cells, and the apoptosis is mediated by the action of caspases and the release of mitochondria/cytochrome-c. In addition, the strength of the apoptosis-inducing effect differed among these pesticides, with the: thiram > ziram > maneb > carbaryl.

## 5. Conclusions

The present study indicates that carbamate pesticides can induce apoptosis in human T cells, and the apoptosis is mediated by the activation of caspases and the release of mitochondrial cytochrome-c.
